# Survival of patients with structurally-grouped *TP53* mutations in ovarian and breast cancers

**DOI:** 10.18632/oncotarget.4080

**Published:** 2015-06-22

**Authors:** Brandon-Luke L. Seagle, Kevin H. Eng, Monica Dandapani, Judy Y. Yeh, Kunle Odunsi, Shohreh Shahabi

**Affiliations:** ^1^ Department of Obstetrics, Gynecology and Reproductive Sciences, Western Connecticut Health Network, Danbury, CT, USA; ^2^ Department of Biostatistics and Bioinformatics, Roswell Park Cancer Institute, Buffalo, NY, USA; ^3^ Department of Gynecologic Oncology, Roswell Park Cancer Institute, Buffalo, NY, USA; ^4^ Division of Gynecologic Oncology, Department of Obstetrics and Gynecology, Prentice Women's Hospital, Northwestern University Feinberg School of Medicine, Chicago, IL, USA

**Keywords:** ovarian neoplasms, breast neoplasms, *TP53* gene, mutation, biological markers

## Abstract

The objective of this study was to determine if ovarian cancer patients with a *TP53* mutation grouped by location of the mutation within the p53 protein structure exhibit differential survival outcomes. Data from patients with high grade serous ovarian cancer (HGS OvCa) (*N* = 316) or breast cancer (BrCa) (*N* = 981) sequenced by The Cancer Genome Atlas (TCGA) was studied by Kaplan-Meier and Cox proportional hazards survival analysis. A *TP53* DNA binding domain (BD) missense mutation (MM) occurred in 58.5% (185/316) of HGS OvCas and 16.8% (165/981) of BrCas. Patients with a *TP53* DNA BD MM grouped by structural location had significantly different overall survival (OS) and progression free survival (PFS). Median OS (months) of HGS OvCa patients by structural group were: Sheet-loop-helix stabilizers, 31.1; DNA minor groove residue R248, 33.6; Wild-type, 34.2; all other MMs, 44.5; DNA major groove residues, 84.1, and zinc ion coordinating residues, 87.0 (log-rank *p* = 0.006). PFS of DNA major groove MM cases was longer than *TP53* wild-type cases (19.1 versus 10.1 months, log-rank *p* = 0.038). HGS OvCa and BrCa patients with structurally-grouped *TP53* DNA BD MMs have different survival outcomes.

## INTRODUCTION

Ovarian cancer is the deadliest gynecologic malignancy, with a 44.6% overall 5-year survival rate and a 1% lifetime mortality rate among American women [1]. High grade serous ovarian cancer (HGS OvCa) is the most common histologic type of ovarian cancer [1]. Surgical staging and cytoreduction followed by adjuvant platinum-taxane chemotherapy is standard management of HGS OvCa [2]. Ovarian cancer was among the first cancers studied by The Cancer Genome Atlas (TCGA) Research Network with multi-dimensional genomic, expression and epigenetic tumor analyses [3]. TCGA's objective in analyzing ovarian cancer was to “identify molecular abnormalities that influence pathophysiology, affect outcomes and constitute therapeutic targets” [3].

Ninety-six percent of 316 HGS OvCa tumors sequenced by TCGA harbored a *TP53* mutation [3]. *TP53* is the most commonly mutated tumor suppressor in human cancers [4]. A large variety of *TP53* mutations including in-frame and frameshift insertions and deletions, missense and nonsense mutations, and splicing alterations are common in human cancers including ovarian cancers [3–6]. Tumor cell aneuploidy and somatic copy number alterations (CNAs) ranging from homozygous deletion to amplification of individual genes are frequently observed in HGS OvCas [3]. Fifteen cases of *TP53* wild-type HGS OvCa had significantly decreased overall survival (OS) and progression free survival (PFS) compared to *TP53* mutant cases [7]. *TP53* missense mutations (MMs) result in a single amino acid substitution in the p53 protein and are the most common oncogenic *TP53* mutations in ovarian cancers [6]. Some MMs may result in gain-of-function p53 activity associated with increased p53 expression [6–9]. Two reports analyzed *TP53* mutant HGS OvCa TCGA cases grouped by gain- or loss-of-function determinations [8, 9]. One study showed no significant differences in OS or PFS comparing gain-of-function versus other mutant *TP53* cases [8]. The second study reported decreased PFS (HR 1.60, *p* = 0.015) among patients with a *TP53* gain-of-function mutation [9].

The three-dimensional crystal structure (PDB file: 1TUP) of the human p53 tumor suppressor DNA binding domain (DNA BD) bound to DNA describes the intra- and intermolecular interactions of p53 amino acids that are most commonly mutated in human cancers [10]. The p53 protein has three domains: the N-terminal transactivation domain, the central DNA BD, and the C-terminal oligomerization domain [9]. Most oncogenic *TP53* MMs occur in the DNA BD [4, 10]. The DNA BD tertiary structure is divided into several substructures, including three loops (L1, L2, L3) and a sheet-loop-helix (SLH) motif that form the DNA binding interface [10]. An appealing approach is to group HGS OvCa patients by the location of each patient's *TP53* MM within the DNA BD tertiary structure. The objective of this study was to determine if structurally-grouped HGS OvCa patients with a *TP53* DNA BD MM experience differential survival outcomes.

## RESULTS

### Ovarian cancer patients with structurally-grouped *TP53* mutations have different survival outcomes

**Figure 1:**
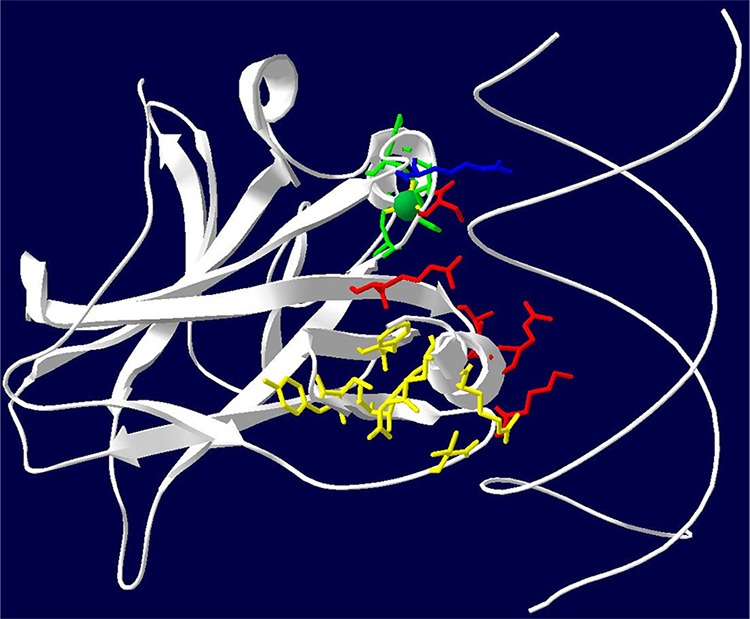
Model of the p53 DNA binding domain structural groups Three-dimensional ribbon diagram of the p53 DNA BD (White) and wire diagram of the DNA double helix backbone. Red: residues that interact directly with the DNA major groove. Blue: R248 residue that interacts directly with the DNA minor groove. Yellow: SLH motif stabilizing residues that support the tertiary structure opposite of the DNA major groove binding interface. Green: Zinc ion and zinc ion coordinating residues that stabilize part of the DNA binding interface.

A *TP53* mutation was identified in 94.6% (299/316) of HGS OvCa TCGA cases. Three cases had two *TP53* mutations. One additional case had homozygous deletion of the *TP53* gene. Sixteen tumors had wild-type *TP53*. A *TP53* MM in the p53 DNA BD occurred in 58.5% (185/316) of all HGS OvCa cases. Structurally-informed groupings of *TP53* DNA BD MMs were determined by the three dimensional protein structure based on the authors’ primary descriptions of residues frequently mutated in human cancers [10]. The direct intermolecular interaction networks of the most frequently mutated residues with neighboring elements of the p53-DNA complex were used to establish structural groups (Table 1) [10]. Initial Kaplan-Meier (KM) survival analysis included all structural groups defined by reference [10] as listed in Table 1. Some structurally-informed groups (hydrophobic core residues, distal loop stabilizers, L2/L3 stabilizers, and L3 group residues) were without significant overall survival (OS) differences (Table 2). These four non-significant structurally-related groups were combined with the other MMs in the DNA BD (Table 2, Reduced groups). Four structurally-related groups (DNA major groove residues, DNA minor groove residue R248, zinc coordinating residues, and sheet-loop-helix (SLH) stabilizing residues) did have significant overall survival (OS) differences by initial KM analysis (Table 2). Stratification of KM analysis by cytoreduction, as well as exclusion of suboptimally cytoreduced cases, increased statistical significance (Table 2). A three-dimensional model of the p53 DNA BD colored by amino acids of each reduced structural group shows that significant structural groups are located within p53 at sites important for its interaction with DNA. Residues of significant structural groups either directly interact with DNA or compose tertiary structures such as the SLH motif and zinc ion binding site that stabilize the p53-DNA interaction (Figure 1). A list of all p53 DNA BD amino acid positions by significant structural group classification is provided (Supplementary Table 1).

**Table 1:** Structurally-related amino acid groups of the p53 DNA binding domain

Structural groups [10]	Amino Acid (single letter code/position number)
DNA major groove interacting residues	R273, S241, A276, R280, C277, R283, K120
DNA minor groove interacting residues	R248
Zinc ion coordinating residues	C176, H179, C238, C242, P177^a^
Sheet-loop-helix (SLH) motif stabilizers	R282, F134, T125, Y126, S127, E286, T118
Loop 2/Loop 3 interaction stabilizers	R175, M237, P191, S183
Distal loop residues	Y220, V157, P151
Loop 3 stabilizers	R249, G245, H162, W163, M246
Hydrophobic core residues	I195, C141, V143, V197, Y234, Y236, F270, F109, L111, L145, V218, T230, I232, I255, L257

a Proline 177 is included with the zinc coordinating residues because it is adjacent to C176. Proline is an amino acid with restricted backbone geometry. Changing P177 to another residue may affect the distance of C176 from the zinc ion. OS and PFS of a single case of MM at P177 were consistent with the group of direct zinc ion coordinating residues. Inclusion of the P177 MM had a negligible affect on calculated statistical significance during KM analysis.

**Table 2:** DNA binding domain *TP53* missense mutations grouped by tertiary structure

High grade serous ovarian cancers								
	All cases				Optimally cytoreduced cases			
Structural groups	*N*	OS	*N*	PFS	*N*	OS	*N*	PFS
Hydrophobic core	20	47.7	18	14.6	15	42.9	13	14.0
Distal loop	17	48.3	16	14.5	12	34.4	11	15.4
L2/L3	10	39.1	10	14.7	8	39.1	8	17.8
L3	13	43.3	10	19.4	4	45.1	4	38.0
Major groove	29	57.3	27	19.1	20	84.1	20	19.1
Minor groove	16	36.1	16	14.1	13	33.6	12	17.6
Other MMs	58	44.5	43	16.1	42	44.1	32	15.4
SLH stabilizer	9	31.1	9	11.2	9	31.1	9	11.2
Wild-type	16	30.9	14	10.1	14	34.2	14	10.1
Zinc binder	13	87.0	10	34.4	8	87.0	7	20.7
*p*-value	0.048 (0.036^a^)		0.038 (0.032^a^)		0.020		0.180	
Reduced groups	*N*	OS	*N*	PFS	*N*	OS	*N*	PFS
Major groove	29	57.3	27	19.1	20	84.1	20	19.1
Minor groove	16	36.1	16	14.1	13	33.6	12	17.6
Other MMs	118	47.4	97	16.1	81	44.5	68	16.1
SLH stabilizer	9	31.1	9	11.2	9	31.1	9	11.2
Wild-type	16	30.9	14	10.1	14	34.2	14	10.1
Zinc binder	13	87.0	10	34.4	8	87.0	7	20.7
*p*-value	0.007		0.038		0.006		0.061	
*All follow-up data truncated at 60 months*								
Reduced groups	*N*	OS	*N*	PFS	*N*	OS	*N*	PFS
Major groove	29	57.3	27	19.1	20	> 60	20	19.1
Minor groove	16	36.1	16	14.1	13	33.6	12	17.6
Other MMs	118	47.4	97	16.1	81	44.5	68	16.1
SLH stabilizer	9	31.1	9	11.2	9	31.1	9	11.2
Wild-type	16	30.9	14	10.1	14	34.2	14	10.1
Zinc binder	13	> 60	10	34.4	8	> 60	7	20.7
*p*-value	0.007		0.038		0.006		0.061	
Risk groups	*N*	OS	*N*	PFS	*N*	OS	*N*	PFS
Lower risk	42	> 60	37	19.8	28	> 60	27	19.9
Typical risk	118	47.4	97	16.1	81	44.5	68	16.1
Higher risk	25	33.6	25	11.2	22	33.6	22	11.2
Wild-type	16	30.9	14	10.1	14	34.2	14	10.1
*p*-value	0.001		0.014		0.002		0.023	

Breast cancers				
*All follow-up data truncated at 60 months*				
Reduced groups	*N*	OS	*N*	PFS
Major groove	15	> 60	14	> 60
Minor groove	5	21.4	5	19.0
Other MMs	104	> 60	94	> 60
SLH stabilizer	7	51.4	3	> 60
Zinc binder	13	> 60	12	> 60
*p*-value	0.153		0.435	
Risk groups	*N*	OS	*N*	PFS
Lower risk	28	> 60	26	> 60
Typical risk	104	> 60	94	> 60
Higher risk	12	51.4	8	> 60
*p*-value	0.085 (0.033^b^)		0.917	

OS: median overall survival (months), PFS: median progression free survival (months), L2/L3:Loop 2/Loop3 interaction stabilizers, L3: Loop 3 stabilizers, MM: missense mutation, SLH: sheet-loop-helix.

a Log-rank (10 groups) with Kaplan-Meier analysis stratified by cytoreduction status.

b From difference of likelihood ratios between Cox proportional-hazards regression models with or without Risk groups.

**Figure 2:**
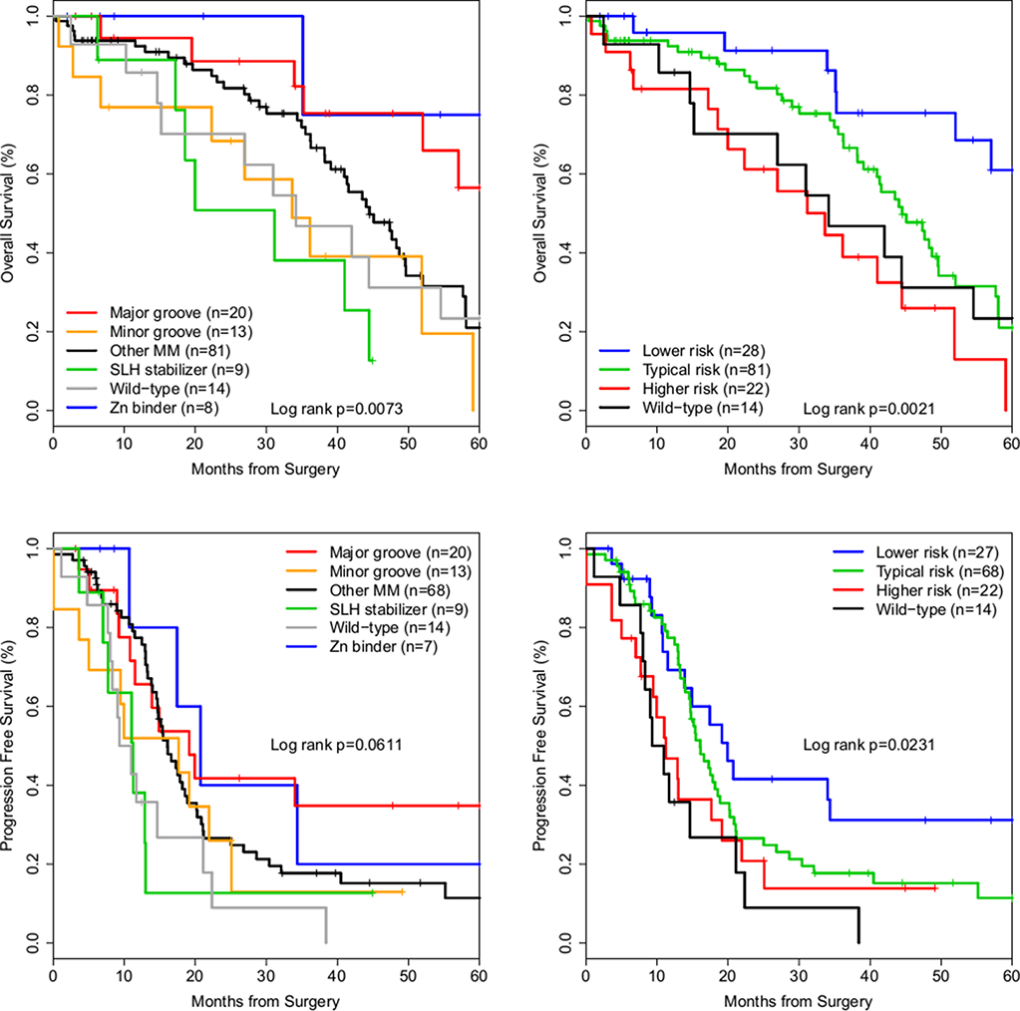
Kaplan-Meier survival curves of *TP53* missense mutation structural groups in ovarian cancer Optimally cytoreduced cases with a *TP53* DNA BD missense mutation. Top row: Overall survival curves. Bottom row: Progression free survival curves. Left column: Cases grouped by reduced structural groups. Right column: Cases grouped by risk groups.

Median OS of optimally cytoreduced cases, in months, comparing reduced structural groups were: SLH stabilizers, 31.1; Wild-type, 34.2; Minor groove residue R248, 33.6, Other MMs, 44.5; Major groove residues, 84.1, and Zinc binders, 87.0 (log-rank *p* = 0.006). Progression free survival (PFS) of Major groove MMs was significantly longer than Wild-type cases (19.1 versus 10.1 months, log-rank *p* = 0.038), but lost significance with exclusion of suboptimally cytoreduced cases (*p* = 0.061). To decrease biasing due to a small number of long-surviving individuals, data was truncated at 60 months and the analysis was repeated, producing similar and significant survival differences (Table 2 and Figure 2). Lastly, groups with similar survival outcomes were combined into Typical risk (Other MM), Higher risk (SLH stabilizers and Minor groove) and Lower risk (Major groove and Zinc binders) groups, and KM survival analysis produced significant differences in OS and PFS (Table 2 and Figure 2).

**Figure 3:**
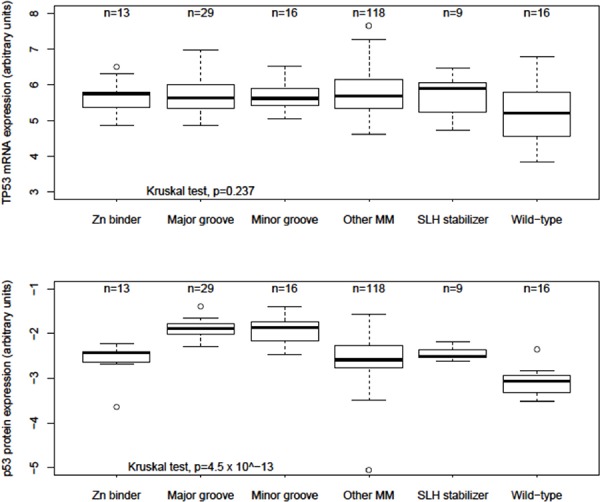
Relative *TP53* mRNA and p53 protein expression among structural groups in ovarian cancer Box and whisker plots of *TP53* mRNA and p53 protein array relative expression levels reported by The Cancer Genome Atlas. Boxes represent 25th to 75th percentile values; dark black lines are median values; whiskers mark 95th percentile range; circles are outlier values.

Age at diagnosis, surgical stage, histologic grade, platinum sensitivity status, *TP53* CNAs, *BRCA* status, and total number of tumor mutations were not significantly different between the reduced structural groups (Table 3). Compared to Other MMs, *TP53* mRNA expression was not significantly different between structural groups, but was significantly decreased among *TP53* Wild-type cases (Wilcoxon test *p* = 0.014) (Figure 3). However, compared to Other MMs, p53 protein expression was significantly increased among Major (Wilcoxon test *p* = 2.8 × 10^−8^) and Minor groove (Wilcoxon test *p* = 2.8 × 10^−6^) groups and decreased among *TP53* Wild-type cases (Wilcoxon test *p* = 9.3 × 10^−6^) (Figure 3). Other MMs, Zn binders and SLH stabilizers had similar p53 protein expression. Additionally, no significant differences in OS or PFS were observed by KM analysis of all or optimally cytoreduced DNA BD MM cases classified by mutation type, secondary structure, *TP53* CNA, truncating versus non-truncating mutations, or among the six most frequently observed hotspot MMs in TCGA cases (Supplementary Table 2), consistent with analyzes previously reported [8].

**Table 3:** Characteristics of optimally cytoreduced DNA binding domain *TP53* missense mutation cases by reduced structural groups

	Major groove	Minor groove	Other MM	SLH stabilizer	Wild-type	Zinc binder	*p*-value
*Age*	58.6	61.1	59.3	55.1	65.5	49.1	0.111^a^
*Stage*							0.356^a^
IIA/B/C	1	0	3	0	0	0	
IIIA/B	2	2	4	1	1	0	
IIIC	15	7	63	5	10	5	
IV	2	4	11	3	3	3	
*Grade*							0.209^a^
G2	1	0	8	0	3	2	
G3	18	13	73	7	10	6	
*Platinum status*							0.114^b^
Sensitive	10	6	40	4	4	4	
Resistant	5	4	13	3	7	0	
*TP53 copy number alteration*							0.820^a^
Gain	1	2	8	0	NA	1	
Heterozygous loss	15	9	59	6	NA	7	
Diploid	4	2	14	3	NA	0	
*Total tumor mutations*	46.5	38.0	49.8	41.0	29.0	35.0	0.802^a^
*BRCA1/2 germline mutation*	6/29	3/16	18/118	1/9	3/13	2/16	0.914^b^

All reported values are exact counts or median values.

a Kruskal-Wallis test

b Fisher's Exact test

Unadjusted Cox proportional-hazards regression hazard ratios (HR) with 95% confidence intervals (CI) for death of patients who underwent optimal cytoreduction and were assigned to groups SLH stabilizer, Minor groove, Wild-type, and Other MMs compared to the Major groove group are HR 6.0 (2.0–17.6), HR 4.1 (1.5–11.2), HR 3.1 (1.2–8.0), and HR 2.5 (1.1–5.5), respectively (likelihood ratio test *p* = 0.007). Each individual group comparison is significant with *p*-values ranging from 0.001–0.029. To adjust for potential cofounding covariates, and including only covariates with greater than 90% data completeness in TCGA datasets for DNA BD MMs cases, multiple multivariate Cox proportional-hazards regression models were tested for covariates age, surgical stage, histologic grade, *TP53* CNA, *TP53* mRNA expression level, and total number of tumor mutations to determine the best regression model. *TP53* CNA gain (*p* = 0.354) and mRNA expression (*p* = 0.949), as well as total number of tumor mutations (*p* = 0.060), were insignificant in the models. Surgical stage (*p* = 0.996) and histologic grade (*p* = 0.854) were also insignificant covariates. Since most tumor mutations are somatic, an interaction term between age and total mutations was also tested and was insignificant (*p* = 0.739). The most significant (likelihood ratio test *p* = 0.0003) regression model included only covariates age (*p* = 0.030), a variable for the presence or absence of a germline *BRCA1* or *BRCA2* mutation (*p* = 0.062), and a log transformation of the total number of tumor mutations (*p* = 0.156). According to this model, HGS OvCas with a *TP53* MM mapping to the SLH group, Minor groove group, or *TP53* Wild-type tumors had HRs (95% CI) for death, compared to the Major groove group, of HR 6.7 (2.2 − 20.1), HR 3.7 (1.3 − 10.2), and HR 3.3 (1.2 − 8.7), respectively, with individual comparison *p*-values ranging from 0.0008–0.019. The difference in survival between the Major groove and Other MMs group was no longer significant (HR 2.1 (0.9 − 4.9), *p* = 0.071).

Cox proportional-hazards regression with covariates age, *BRCA* status, and total number of tumor mutations was also performed by the reduced and risk groups after follow-up data was truncated at 60 months. Among the reduced structural groups, SLH group, Minor groove group, and *TP53* Wild-type tumors had HRs (95% CI) for death, compared to the Major groove group, of HR 6.2 (2.0 − 19.2), HR 3.3 (1.2 − 9.4), and HR 2.9 (1.0 − 8.4), respectively, with individual comparison *p*-values ranging from 0.002–0.043 (overall regression, likelihood ratio test *p* = 0.00075). Age was the only significant covariate (*p* = 0.018). *BRCA* status was less significant in the model after data truncation (*p* = 0.172). The most significant regression model (likelihood ratio test *p* = 0.00036) was of the risk groups, with increased HRs for death compared to the Lower risk group for Typical risk patients (HR 2.3 (1.0 − 5.1) *p* = 0.044), Wild-type patients (HR 3.4 (1.2 − 9.2), *p* = 0.018), and Higher risk patients (HR 4.8 (2.0 − 11.9), *p* = 0.0006). Only age was a significant covariate (*p* = 0.028).

### Differential survival outcomes of patients with structurally-grouped *TP53* mutations were reproduced in a breast cancer validation cohort

**Figure 4:**
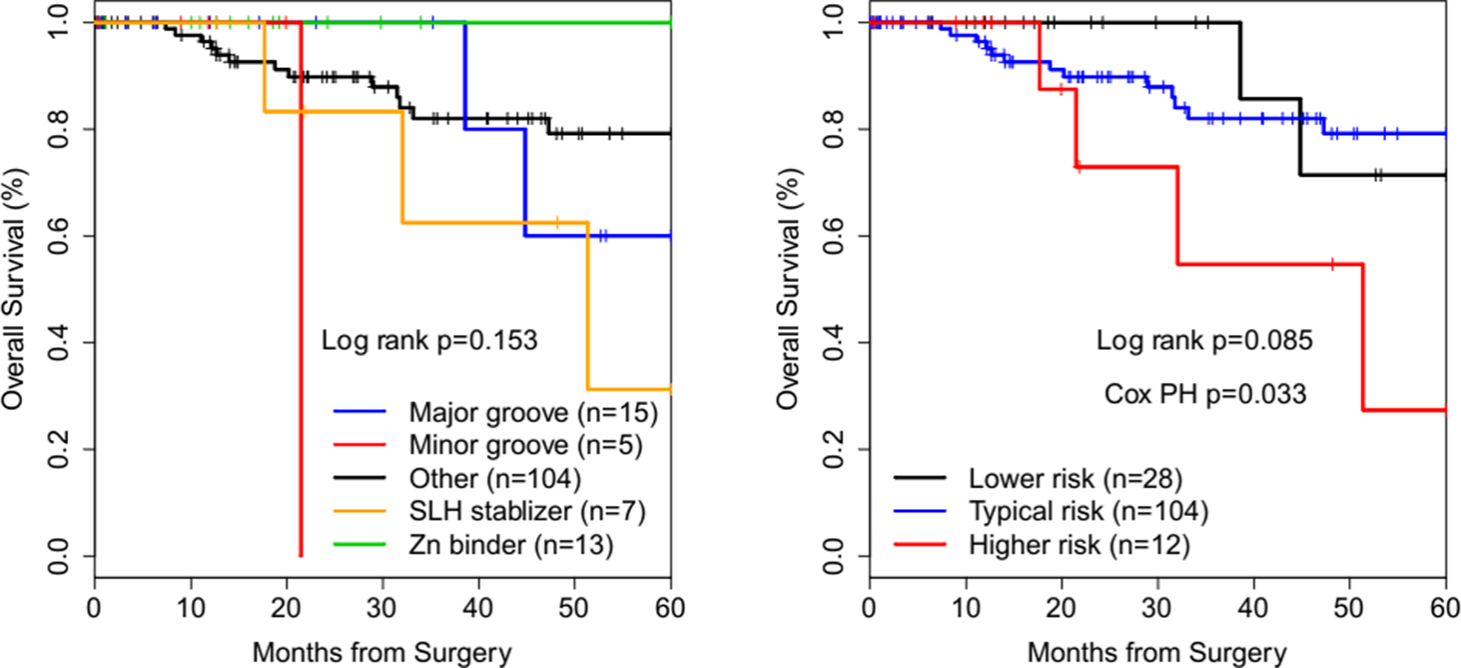
Kaplan-Meier survival curves of *TP53* missense mutation structural groups in breast cancer Cox PH – Cox proportional-hazards regression. *p*-value refers to significance of the contribution of risk groups to the regression model by taking the difference in likelihood ratios between two regression models, one with and one without including risk groups as a covariate.

The same strategy described above for analysis of HGS OvCa was applied to BrCa cases, using identical criteria for assignment of cases to reduced structural (Table 1) and risk groups. Empirical KM analysis showed trends towards statistical significance for BrCas (Figure 4 and Table 2). Follow-up data was also truncated at 60 months. TCGA BrCas are clinically a more heterogeneous population (by stage and histology) than TCGA HGS OvCas. A Cox proportional-hazards model (likelihood ratio text *p* = 0.0005) of reduced structural groups with significant covariates age (HR 1.1 (1.0 − 1.2 *p* = 0.011) and invasive ductal (ICD-0-3 code 8500/3) histologic type (HR 0.06 (0.006 − 0.70), *p* = 0.024) demonstrated, compared to the Major groove group, significantly decreased survival of patients in the Minor groove group (HR 132.7 (1.7 − 1.0 × 10^4^ *p* = 0.028), Other MM group (HR 39.0 (2.5 − 609.7) *p* = 0.009), or SLH group (HR 297.1 (8.8 − 1.0 × 10^4^ *p* = 0.002).

For risk groups, a highly significant (likelihood ratio test *p* = 0.0002) Cox proportional-hazards model included statistically significant covariates age (HR 1.1 (1.0 − 1.2) *p* = 0.004), stage IV (HR 173.8 (9.1 − 3325.5) *p* = 0.0006), and invasive ductal (ICD-0-3 code 8500/3) histologic type (HR 0.06 (0.006 − 0.68) *p* = 0.023). Risk groups contributed significantly to the OS model (likelihood ratio test *p* = 0.033) (Figure 4). Compared to the Lower risk group, HRs for death for Typical risk (HR 42.6 (2.7 − 669.4) *p* = 0.008) and Higher risk (HR 269.5 (8.4 − 8688.0) *p* = 0.002) groups were significantly increased.

Using TCGA data on molecular subtypes of breast cancer among these cases, a regression model including covariates for estrogen, progesterone, and HER2 receptor positivity/negativity was developed (likelihood ratio test *p* = 0.00004). In this model, age (HR 1.3 (1.1 − 1.5) *p* = 0.005), invasive ductal (ICD-0-3 code 8500/3) histologic type (HR 0.02 (0.0006 − 0.40) *p* = 0.012), and progesterone receptor positivity (HR 0.01 (0.0002 − 0.96) *p* = 0.048) were significant covariates. Compared to the Lower risk group, HRs for death for Typical risk (HR 9.8 × 10^5^ (4.1 × 10^2^ − 2.4 × 10^9^) *p* = 0.0005) and Higher risk (HR 1.3 × 10^6^ (3.7 × 10^2^ − 4.9 × 10^9^) *p* = 0.0008) groups were significantly increased. TCGA-reported PAM50 molecular subtype information was available for some *TP53* MM cases analyzed by our study [11–13]. There were no significant differences in PAM50 molecular subtypes between *TP53* structural mutant risk groups (Fisher's exact test *p* = 0.804, Supplementary Table 3). The Cox regression model including PAM50 subtypes included only 82 cases due to limited numbers of cases with reported PAM50 subtype. In the PAM50 subtype adjusted model (likelihood ratio test *p* = 0.0001), age (HR 1.6 (1.1 − 2.4) *p* = 0.022), invasive ductal (ICD-0-3 code 8500/3) histologic type (HR 4.1 × 10^−4^ (8.5 × 10^−7^ − 0.2) *p* = 0.013), and Luminal A subtype (HR 316.8 (2.3 − 4.4 × 10^4^) *p* = 0.022) were significant covariates. Compared to the Lower risk group, HRs for death for Typical risk (HR 1.4 × 10^4^ (19.4 − 1.1 × 10^7^) *p* = 0.005) and Higher risk (HR 1.9 × 10^5^ (1.5 − 2.5 × 10^10^) *p* = 0.042) groups were significantly increased. Therefore, *TP53* mutation structural mutant risk groups were more significantly associated with overall survival among breast cancer patients with a *TP53* DNA BD MM than progesterone, estrogen or HER2 receptor status, or PAM50 molecular subtype.

## DISCUSSION

Patients with ovarian or breast cancer and a *TP53* MM within different tertiary substructures of the p53 DNA BD may have significantly different OS or PFS. Structural groups significantly associated with OS of HGS OvCa and BrCa patients map to areas of the p53 DNA BD that directly interact with DNA or that stabilize the DNA interaction surface. Presumably by altering interaction of p53 with its DNA recognition sequence, mutated residues in the significant structural groups may cause different p53 functionality compared to wild-type p53 residues at the same locations. Laboratory studies may be needed to explain any structural-functional associations broached by these findings. HGS OvCa patients with a *TP53* R248 mutation have decreased survival, consistent with observations that ovarian cancer cells with a R248 mutation are relatively platinum and taxane chemoresistant [9]. The most frequently observed mutation in the SLH motif is of R282. Patients with Li-Fraumeni syndrome and a germline R282 mutation were shown to have a significantly earlier age of diagnosis (median 13 years old) of their first cancer compared to patients with other germline *TP53* mutations [14].

We show that breast cancers with a *TP53* missense mutation of a residue that coordinates the zinc ion had increased survival among TCGA cases (Figure 4). Three previous studies reported that breast cancers with *TP53* mutations in the misnamed “zinc domain” had either no difference in survival outcomes or worse survival [15–17]. All three papers defined in the zinc domain as the L2 and L3 loops rather than specifically analyzing residues that directly coordinate the zinc ion [15–17]. These studies included multiple *TP53* mutation types such as nonsense and frameshift mutations [15–17]. One study that explicitly defined all mutations included in the analysis did not include any missense mutations that bind the zinc ion [17]. The study with the greatest number of *TP53* mutations included residues that interact with DNA or contribute to the SLH motif [16]. The authors reported that when analyzed separately the residues that interact with DNA or contribute to the SLH motif were non-significantly associated with decreased OS (*p* = 0.11), which may be consistent with our findings. Due to small case numbers these studies were unable to compare structural groups defined by intermolecular interactions within the tertiary structure of the p53 DNA BD. Our finding that progesterone receptor (PR) positivity was associated with significantly decreased HR for death among the TCGA cohort of *TP53* DNA BD MM BrCa patients is consistent with a previous report that *TP53* mutant/PR negative BrCa patients had decreased survival compared to *TP53* mutant/PR positive patients [18]. This study also showed that BrCa with a R248W have decreased OS compared to other common hot spot mutations and that estrogen receptor status did not impact survival among *TP53* mutant BrCa patients, both consistent with our findings [18].

The potential clinical utility of our findings is also of interest. Currently, the *TP53* mutation harbored by a primary ovarian tumor is not determined for clinical purposes and has no role in patient management when determined during a research protocol. Additional clinically-annotated mutational datasets are needed to validate if the *TP53* mutation status and particular *TP53* mutations of primary ovarian tumors are clinically prognostic. Determining associations of *TP53* mutations with clinical chemotherapy resistance is also important. Given that most HGS OvCas contain a *TP53* mutation, one strategy to efficiently investigate if particular mutations are associated with chemoresistance would be to perform targeted *TP53* exon sequencing of tumors that are also analyzed by *in vitro* chemotherapy sensitivity assays.

Limitations of this study are limited case numbers and incompleteness of data (such as progression free survival time, platinum sensitivity status, and PAM50 subtypes) in existing clinically-annotated datasets. For instance, there is not enough statistical power to reach significance for differences in *BRCA* status between the structural-related mutation groups or show significance of *BRCA* status in multivariate Cox regression of structural-related mutation groups. However, *BRCA* status among these groups likely plays a role, especially among very long surviving individuals. *BRCA* status became less significant in multivariate regression after follow-up data was truncated at 60 months. Similarly, PAM50 subtype is not reported for enough patients to confidently determine if *TP53* structurally-related mutational groups are associated with particular PAM50 subtypes. Our finding that Luminal A subtype was associated with increased HR for death is contradictory to previous reports that Luminal A patients have improved survival [12, 13]. Limited data (*N* = 8) reported that patients with Luminal A and a *TP53* mutation may have decreased breast cancer specific survival compared to Luminal A tumors without a *TP53* [19]. Our analysis included 17 patients with Luminal A subtype, all of whom have a *TP53* mutation. It may be that among patients with a *TP53* DNA BD MM, Luminal A subtype is significantly associated with decreased OS. However, this finding is not consistent with existing literature and analysis of a larger cohort is needed to test this association [19].

TCGA is the only publically available data source for our analysis in ovarian cancer. Combined with the inherent genetic heterogeneity of HGS OvCa, limited case numbers and data incompleteness make meaningful targeted analysis of TCGA datasets difficult. Even when performing one-dimensional mutational analysis of the single gene, *TP53*, the heterogeneity of HGS OvCa is reflected by the large variety of mutations observed, which subdivides the available cases into smaller comparison groups. These circumstances are fertile ground for more complex multi-dimensional analyses of available datasets, as is exemplified by TCGA's own expression profiling of HGS OvCa tumors [3]. Developing practical, testable hypotheses or clinically applicable findings from multi-dimensional genomic, expression and epigenetic analyses remains a major challenge to translating findings of such analyses into practice. Finally, differences in overall survival observed here could be related to differences in treatment response after disease recurrence.

The appeal of the structurally-driven approach applied here is that it begins with the concept that if different MMs have any clinical significance compared to each other, then the differences may originate from the protein product of these mutations. Second, in order to group MMs into larger groups than single codon groups without over-aggregating cases and loosing resolution of potentially significant differences between subsets of MMs, the p53 protein structure was used as a map for grouping cases with MMs. This simple idea leads to assignment of patient groups with large and significant differences in overall survival, albeit deserving of reserved confidence in the ability to adjust for covariates given small case numbers and data incompleteness. Validation of our findings with additional clinically-annotated tumor genomic datasets is required. Finding similar OS differences among structurally-related risk groups in TCGA BrCas provided limited, independent validation. Testable and clinically relevant hypotheses are generated by this study. We used a structurally-driven approach to single-gene mutational analysis to discover significantly different *TP53* mutational groups in high grade serous ovarian cancer.

## MATERIALS AND METHODS

### Data collection from The Cancer Genome Atlas

TCGA cases of HGS OvCa (*N* = 316) and breast cancer (BrCa) (*N* = 981) with donated tumor tissue used for whole exome DNA sequencing were identified using the cBioPortal.org application [18, 19]. For each case, information was extracted from cBioPortal or downloaded from the TCGA Data Portal including the presence or absence of a *TP53* mutation, *TP53* mutation type, genomic and codon locations, *TP53* DNA CNA, relative *TP53* mRNA and p53 protein expression levels, and total number of mutations (considering all genes accessed by whole exome sequencing) in each tumor. Matched clinical data including vital status, recurrence/progression status, OS and PFS times, residual disease after cytoreduction, surgical stage and histologic grade, and platinum sensitivity status for all identified cases was also collected from the cBioPortal.org application [18, 19]. Age at diagnosis was collected from the TCGA global analysis report supplementary information and matched by unique TCGA case identification codes [3]. TCGA reported that HGS OvCa patients were treated by surgical staging and cytoreduction followed by adjuvant platinum (100%) and taxane (94%) combination chemotherapy [3]. One HGS OvCa case received neoadjuvant chemotherapy and is not among the 316 cases that were sequenced.

### Creation of patient groups by structurally-related *TP53* mutations

Cases were initially classified based upon *TP53* mutation type and location within the p53 protein. Cases with a *TP53* MM were then further grouped by information regarding location and intra- and intermolecular interaction patterns of wild-type amino acids at MM locations within the p53 protein DNA BD three-dimensional structure (PDB file: 1TUP) complexed with DNA [10]. Structurally-related amino acid groups were identified based directly on amino acid groups and *TP53* oncogenic hot spot mutation site amino acid interaction descriptions from the original crystal structure report [10] (Table 1). No MMs occurred at any amino acid with an outlier backbone geometry as calculated for 1TUP by a MolProbity Ramachandran plot [20]. *TP53* MM cases were then assigned by codon location to structurally-related groups (Supplementary Dataset 1). The human p53 wild-type DNA BD with amino acids colored by structural group was modeled with the Swiss PDB viewer [21].

Kaplan-Meier survival analysis, Cox proportional-hazards regression, and all statistical testing was performed in the R statistical programing language [22, 23]. Survival outcomes of cases were compared according to mutation type, by p53 protein truncating versus non-truncating mutations, by location in the p53 DNA BD secondary structure simply by amino acid sequence without consideration of tertiary structure and molecular interactions, simply by grouping cases according to the six most frequently observed hot spot MMs, and also by structurally-informed groups (Table 2 and Supplementary Table 2). As indicated in Table 2, some comparisons were repeated with exclusion of suboptimally cytoreduced cases. The survival analysis was repeated with truncation of follow-up data at 60 months to decrease biasing of the results by a small number of unusually long-surviving patients (Table 2). Cytoreduction was considered suboptimal if residual disease after surgical staging as reported by TCGA was > 10 mm. Age at diagnosis, surgical stage, histologic grade, platinum sensitivity status, *TP53* CNA, germline *BRCA* mutation status, and total number of mutations in the tumor, were compared between optimally cytoreduced structurally-related groups using appropriate statistical tests (Table 3). To verify findings from HGS OvCas, survival outcomes of patients with BrCa and a *TP53* DNA BD MM were compared, using the same structurally-related groupings as was applied to HGS OvCas (Table 1 and Supplementary Dataset 2). Cox proportional-hazards regressions were performed to adjust for covariates.

## SUPPLEMENTARY TABLES








