# Supplementary material for: Survival of patients with structurally-grouped TP53 mutations in ovarian and breast cancers
Source: Oncotarget. 2015 Jun 22;6(21):18641–52. doi: 10.18632/oncotarget.4080 (PMC4621916; doi:10.18632/oncotarget.4080)
Supplement: Supplementary file 1 [file oncotarget-06-18641-s001.pdf]

SUPPLEMENTARY TABLES

Supplementary Table 1: Structural group assignment of p53 DNA binding domain residues

Supplemental Table 2. Additional survival analyses. All *p*-values are from a log rank test
Analysis by *TP53* mutation status (all cases):

***TP53* mutations:**	***N***	**Median OS**	***N***	**Median PFS**
Homozygous del	1	NA	1	NA
Frame shift (FS) indel	41	39.6	34	16.8
In frame (IF) indel	9	38.0	7	19.0
Missense	187	47.4	161	16.1
Nonsense	29	43.9	26	17.5
Splice	35	45.8	26	17.5
WT	16	30.9	14	10.1
	p-value:	0.675	0.211	

—Mutation type is not associated with significant survival differences.

Analysis by type of *TP53* mutations (optimally cytoreduced cases):

***TP53* mutations:**	***N***	**Median OS**	***N***	**Median PFS**
Frame shift (FS) indel	23	47.5	22	16.8
In frame (IF) indel	7	45.0	5	19.0
Missense	132	44.5	118	16.1
Nonsense	18	43.9	17	17.5
Splice	20	44.3	14	17.5
WT	14	34.2	14	10.1
p-value:		0.799	0.196	

—Mutation type is not associated with significant survival differences among optimally cytoreduced cases.

Analysis of all cases by cytoreduction status:

**Cytoreduction:**	***N***	**Median OS**	***N***	**Median PFS**
Optimal	214	44.5	190	16.1
Suboptimal	70	38.0	49	14.1
p-value:		0.030	0.181	

—OS is significantly different among all cases grouped by cytoreduction status.

Analysis of all cases by platinum sensitivity status:

Platinum status:	*N*	Median OS	*N*	Median PFS
Sensitive	133	58.0	132	20.2
Resistant	65	32.9	65	9.1
	p-value:	< 0.0001	< 0.0001	

—Platinum status is highly significantly associated with differences in OS and PFS.

Analysis of all cases by *TP53* copy number alteration:

TP53 mutations:	N	Median OS	N	Median PFS
Amplication	2	22.2	2	10.9
Deletion	1	NA	NA	NA
Diploid	49	51.9	43	18.2
Gain	26	35.8	22	14.7
Heterogeneous loss	224	45.1	187	16.3
	p-value:	0.814	0.728	

—No significant difference in survival outcomes by copy number alternations among all cases.

Analysis of all *TP53* mutations that caused or did not cause p53 truncation regardless of cytoreduction (all cases):

Truncation:	*N*	Median OS	*N*	Median PFS
Yes	192	45.1	165	16.1
No	109	44.3	89	17.5
	p-value:	0.890	**0.841**	

—No significant differences by truncating versus nontruncating mutation among all cases.

Analysis of all *TP53* mutations that caused or did not cause p53 truncation (optimally cytoreduced cases only):

Truncation:	*N*	Median OS	*N*	Median PFS
Yes	136	44.5	121	16.1
No	64	44.3	55	17.5
	p-value:	0.971	0.695	

—No significant differences by truncating versus nontruncating mutation among optimally cytoreduced cases.

Analysis of DNA BD MM cases by *TP53* copy number alteration among all cases:

***TP53* mutations:**	***N***	**Median OS**	***N***	**Median PFS**
Amplication	1	NA	1	NA
Diploid	28	51.9	27	14.8
Gain	15	29.0	13	14.7
Heterogeneous loss	141	45.1	118	16.3
	p-value:	0.332	0.673	

—No significant difference in survival outcomes by copy number alternations among DNA BD MM.

Analysis of DNA BD MM cases by *TP53* copy number alteration among only optimally cytoreduced cases:

***TP53* mutations:**	***N***	**Median OS**	***N***	**Median PFS**
Diploid	23	51.9	22	15.4
Gain	12	36.1	11	15.4
Heterogeneous loss	96	44.5	84	16.1
	p-value:	0.756	0.987	

—No significant difference in survival outcomes by copy number alternations among DNA BD MM that were optimally cytoreduced.

DNA BD MM and WT cases by cytoreduction status:

**Cytoreduction:**	**N**	**Median OS**	**N**	**Median PFS**
Optimal	145	44.5	131	15.2
Suboptimal	35	43.3	25	14.1
	p-value:	0.478	0.284	

—No significant difference in survival outcomes by cytoreduction status among DNA BD MM and WT cases.

Analysis of DNA BD MM cases by platinum sensitivity status:

**Platinum status:**	***N***	**Median OS**	***N***	**Median PFS**
Sensitive	89	57.0	88	20.2
Resistant	43	33.6	43	9.0
	p-value:	< 0.0001	< 0.0001	

—Platinum status is highly significantly associated with differences in OS and PFS.

Analysis of optimally cytoreduced DNA BD MM cases by platinum sensitivity status:

Platinum status:	*N*	Median OS	*N*	Median PFS
Sensitive	68	51.9	67	20.2
Resistant	32	35.2	43	7.7
	p-value:	0.0002	< 0.0001	

—Platinum status is highly significantly associated with differences in OS and PFS.

Analysis by DNA BD MM cases with the 6 most frequently observed hotspot mutations in TCGA:

Amino acid:	*N*	Median OS	*N*	Median PFS
175	8	62.1	8	16.3
195	9	47.7	8	14.6
220	11	49.2	10	17.5
245	9	43.3	7	18.0
248	16	36.1	16	14.1
273	21	84.1	21	14.5
p-value:		0.282	0.968	

—No significant survival differences between cases groups by most frequently observed hotspot mutations. Same is seen for optimally cytoreduced cases: OS (p=0.182), PFS (p=0.868).

Analysis of DNA BD MM cases by secondary structure:

Secondary structure:	*N*	Median OS	*N*	Median PFS
Beta sheet	58	47.5	47	16.6
Loop 1	11	57.7	9	11.1
Loop 2	32	49.6	27	17.8
Loop 3	44	43.3	38	16.1
Sheet-loop-helix motif	40	52.4	38	13.9
	p-value:	0.741	0.939	

—No significant survival differences by secondary structural classification of DNA BD MM cases. Same holds true with analysis of only optimally cytoreduced cases: OS (p=0.315), PFS (p=0.905).

Supplementary Table 3:PAM50 molecular subtypes among TCGA breast cancer cases (N = 1104)[a]

	PAM50 Subtype					
**Cases:**	**Unclassified**	**Basal-like**	**HER2-enriched**	**Luminal A**	**Luminal B**	**Normal-like**
Not sequenced	106	3	2	6	6	0
Not a *TP53* DNA BD MM	407	63	32	208	101	6
Lower risk	17	4	5	2	4	1
Typical risk	46	24	18	14	15	1
Higher risk	6	4	1	1	1	0

[a]According to cBioPortal search of TCGA Provisional dataset. PAM50 data from the TCGA Nature 2012 breast cancer clinical data searched using the CGDS-R package.
DNA BD MM: DNA binding domain missense mutation.
